# New Enclosure for *in vivo* Medical Imaging of Zebrafish With Vital Signs Monitoring

**DOI:** 10.3389/fphys.2022.906110

**Published:** 2022-06-29

**Authors:** A. C. M. Magalhães, P. M. M. Correia, R. G. Oliveira, P. M. C. C. Encarnação, I. Domingues, J. F. C. A. Veloso, A. L. M. Silva

**Affiliations:** ^1^ Department of Physics, I3N, University of Aveiro, Aveiro, Portugal; ^2^ CESAM, Department of Biology, University of Aveiro, Aveiro, Portugal

**Keywords:** Zebrafish physiology, non-invasive sensors, vital signs monitoring, zebrafish heart rate, small animal imaging

## Abstract

Lately, the use of zebrafish has gained increased interest in the scientific community as an animal model in preclinical research. However, there is a lack of *in vivo* imaging tools that ensure animal welfare during acquisition procedures. The use of functional imaging techniques, like Positron Emission Tomography (PET), in zebrafish is limited since it requires the animal to be alive, representing a higher instrumentation complexity when compared to morphological imaging systems. In the present work, a new zebrafish enclosure was developed to acquire *in vivo* images while monitoring the animal’s welfare through its heartbeat. The temperature, dissolved oxygen, and pH range in a closed aquatic environment were tested to ensure that the conditions stay suitable for animal welfare during image acquisitions. The developed system, based on an enclosure with a bed and heartbeat sensors, was tested under controlled conditions in anesthetized fishes. Since the anesthetized zebrafish do not affect the water quality over time, there is no need to incorporate water circulation for the expected time of PET exams (about 30 min). The range of values obtained for the zebrafish heart rate was 88–127 bpm. The developed system has shown promising results regarding the zebrafish’s heart rate while keeping the fish still during the long imaging exams. The zebrafish enclosure ensures the animal’s well-being during the acquisition of *in vivo* images in different modalities (PET, Computer Tomography, Magnetic Resonance Imaging), contributing substantially to the preclinical research.

## 1 Introduction

Most preclinical experimental studies are conducted in animal models, traditionally mammals, like rodents. This is mainly due to the homology of the mammalian genome, anatomy, cellular biology, and physiology (Merrifield et al., 2017). However, these models have significant disadvantages, such as the high maintenance cost and complex genomes (Volkoff, 2019). On the other hand, non-mammalian vertebrates, mainly fishes, have genetic, endocrine, and physiological features, brain mechanisms, and essential gut functions similar to humans (Volkoff, 2019). These similarities allow using fishes as models of human diseases (Barbazuk et al., 2000; Kari, Rodeck and Dicker, 2007; Lieschke and Currie, 2007; Feitsma and Cuppen, 2008; Sun et al., 2008; Howe et al., 2013; Jones and Norton, 2015; Meshalkina et al., 2017; Sieber et al., 2019).

Zebrafish have short life cycles, and when compared with mammalian models, they have lower maintenance costs and fewer ethical issues involved in conducting experiments, facilitating genetic manipulation (Kari, Rodeck and Dicker, 2007; Volkoff, 2019) and allowing faster and cheaper large-scale studies when compared with other vertebrate models (Bufkin and Leevy, 2015). Zebrafish also has a unique natural ability to regenerate some tissues, and its genome has already been sequenced, which makes it very attractive to different research areas, such as evolutionary biology, and regenerative medicine (Lieschke and Currie, 2007; Howe et al., 2013; Marques, Lupi and Mercader, 2019). Due to its advantages, zebrafish models are becoming increasingly important and used in preclinical research. There is a clear interest in using and studying this animal model using medical imaging (Yao, Lecomte and Crawford, 2012); however, the imaging systems are rarely used (Browning, 2013), mainly due to the lack of technology and tools oriented to the acquisition of *in vivo* zebrafish images (Koba, Jelicks and Fine, 2013). Furthermore, its small size and physiological requirements make it very challenging and require the development of a dedicated housing capable of keeping the fish still and monitoring physiological parameters to ensure the animal’s welfare during imaging procedures.

Positron Emission Tomography (PET) is a powerful image research tool for many biological research activities. The non-invasive image provided by PET technology and its ability to detect changes in metabolic, biological, chemical, and molecular processes may provide new scientific discoveries and economic advantages in research with zebrafish (Bufkin and Leevy, 2015; Dorsemans et al., 2017). However, only a few studies using PET in zebrafish are reported in the literature (Bufkin and Leevy, 2015; Dorsemans et al., 2017; Jones et al., 2017; Snay, 2017; Tucker et al., 2021). In all of those, the equipment used has been particularly rudimentary for physiological preservation and animal welfare. In these *in vivo* studies, zebrafish were either upright or wrapped in absorbent tissue (Bufkin and Leevy, 2015; Dorsemans et al., 2017; Jones et al., 2017; Snay, 2017; Tucker et al., 2021). These conditions are not natural for the fish, adding undesirable physiological stress.

More recently, new dedicated chambers have been developed ((Koth et al., 2017; Merrifield et al., 2017; Seeger et al., 2022)) that have water circulation ensuring a continuous supply of fresh water and anesthetics. Despite the monitoring of the environmental conditions, these chambers did not monitor the zebrafish’s physiological stability. In addition to being able to monitor the well-being of the fish over time, this information will also be helpful to use as cardiac gating in the various imaging modalities (e.g., Merrifield et al., 2013).

Therefore, to address these challenges, a new dedicated zebrafish enclosure capable of keeping it still and monitoring the animal’s vital signs during the imaging exam is presented, demonstrating the capability to predict critical injuries of the animal and prevent them in time during acquisitions procedures.

## 2 Zebrafish Ethical Principles

The zebrafish used in this study were protected under the directive 2010/63/EU, following the 3Rs principle (Replace, Reduce, and Refine). Therefore, the experimental design was meticulously planned to decrease the chance of errors and unsuccessful experiments; additionally, to avoid unnecessary testing, an extensive literature review, covering a broad number of areas involving the problem, was carried out. Furthermore, the fish’s well-being, including anesthesia administration and recovery, was assessed during the experiments to minimize suffering. Finally, euthanasia was performed according to the methods approved by the competent entities when needed.

## 3 Zebrafish Preparation

The zebrafish used in this work were kept in tanks with proper conditions at the bioterium of the Biology Department of the University of Aveiro. Adult zebrafish were maintained in carbon-filtered water with salt (*Instant Ocean Synthetic Sea Salt*) under a 12:12 h (light: dark) cycle and at 26 ± 1°C. The water was continuously renewed, and physicochemical parameters were controlled (pH 7.5 ± 0.5; conductivity 750 ± 50 µS and dissolved oxygen above 95% saturation).

**FIGURE 1:**
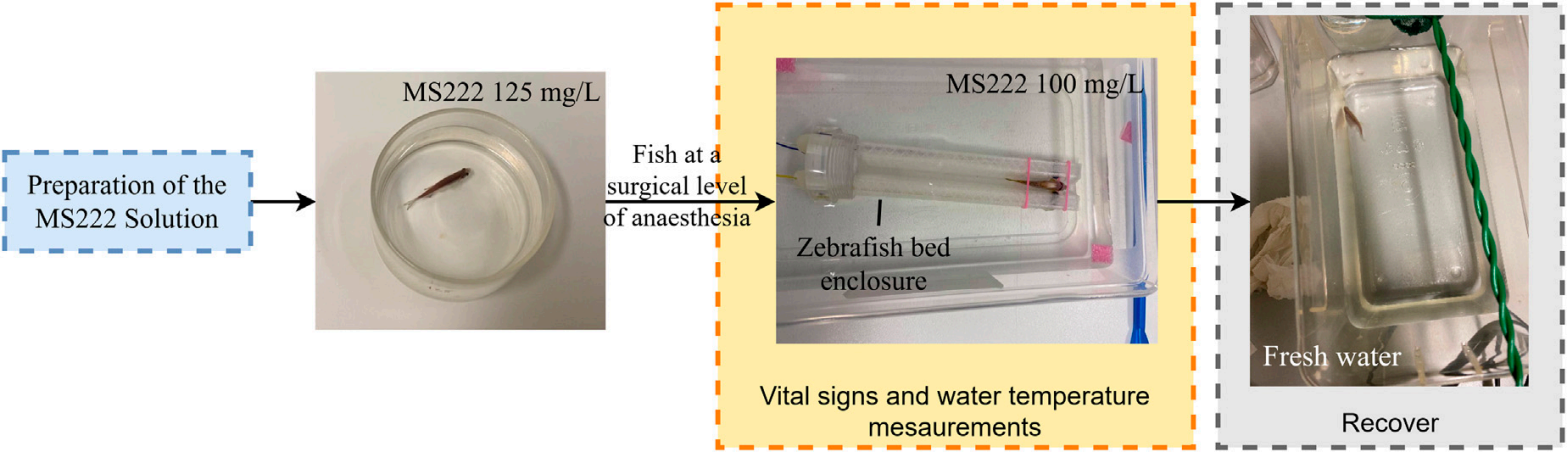
Anesthesia protocol followed in the present work.

The experiments with zebrafish required their immobilization using an anesthetic like *tricaine methanesulfonate* (MS222), which is the most used and well-established for this species (Collymore et al., 2014). The anesthetic works as a muscle relaxant, reducing the muscle action potentials and spontaneous contractions (including sensory inputs and reflexes) (Matthews and Varga, 2012). The protocol followed in the present work to anesthetize zebrafish is described in Figure 1 and was based on the one reported in (Merrifield et al., 2017).

The protocol starts by emerging the zebrafish on a solution of MS222 (125 mg/L) until reaching the surgical level of anesthesia, which can be identified through the loss of equilibrium, no reflex response, and the slowdown of opercular movements. After reaching this level of anesthesia, the zebrafish is transferred to a 100 mg/L of MS222 solution, where it is kept to maintain the anesthesia effect. The anesthetized zebrafish tend to be laid on their back, so we decided to maintain them in this position in the enclosure during our measurements. However, there is no technical limitation in doing the heartbeat measurements with the zebrafish in its natural position when awaked.

## 4 Zebrafish Enclosure

**FIGURE 2:**
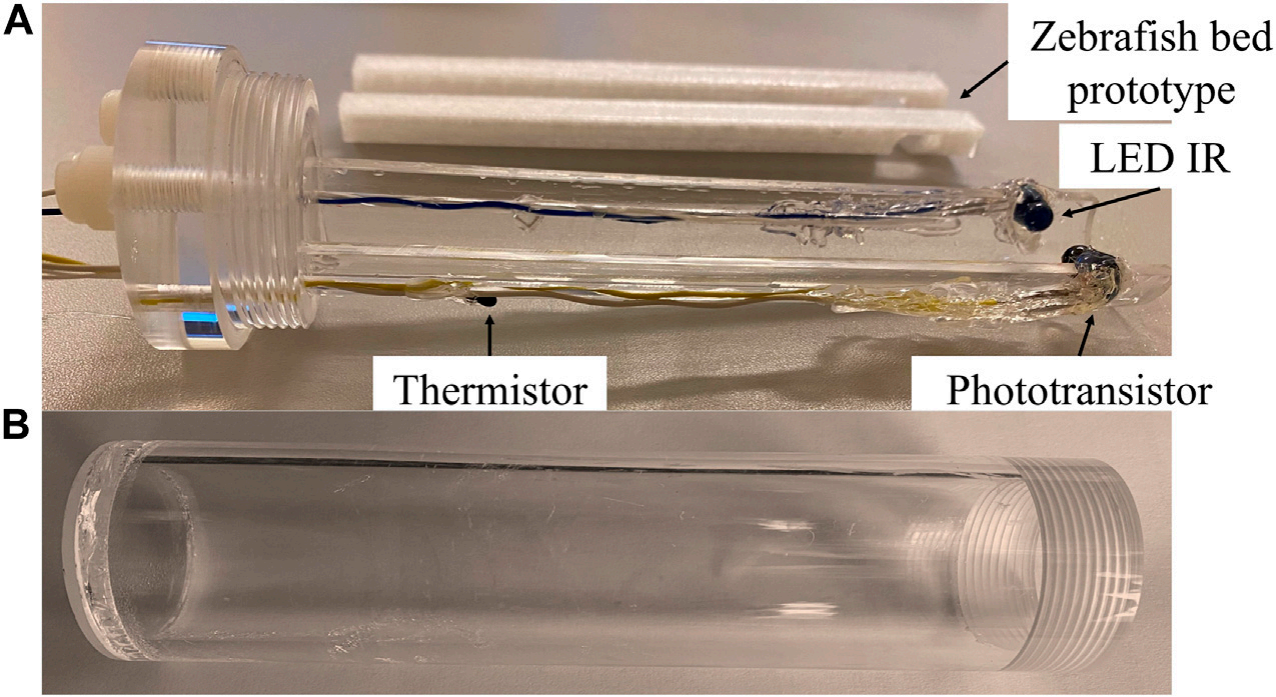
Enclosure prototype developed **(A)** Bed support block to accommodate zebrafish **(B)** Block to seal the container.

The developed enclosure consists in a closed cylindrical chamber made with PMMA (Poly (methyl methacrylate)). The chamber material was chosen due to its transparency, enabling the visualization of the fish and ensuring that the animal does not exhibit signals of stress. In addition, the material has a reduced atomic number (water equivalent), which has less interaction with the radiation during the imaging exam minimizing the image deterioration. The chamber includes inside a bed support to accommodate the zebrafish in a position aligned with the heartbeat sensors. Beyond these sensors, the developed enclosure also contains a thermistor to measure the water temperature (Figure 2).

The proposed bed configuration intends to improve the zebrafish placement in a horizontal position, preventing the fish from moving as well as allowing an effortless sealing of the container when filled with the anesthetic solution.

**FIGURE 3:**
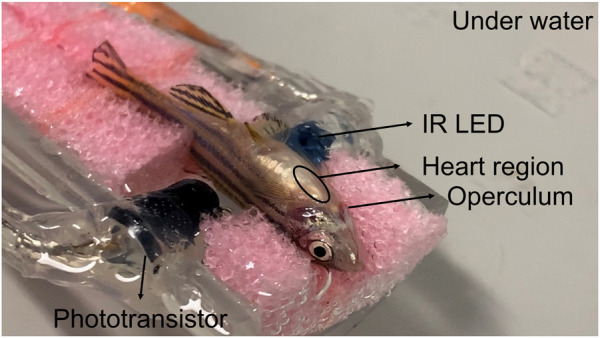
Zebrafish in the bed with a sponge to improve immobilization and positioning of zebrafish.

Additionally, a sponge was added to the support block of the zebrafish’s bed to immobilize it more efficiently (Figure 3). Since the opercula need to move freely and without the interference of the immobilization material, the sponge has a broad aperture in the zebrafish’s head region. In order to ensure that the animal stays still during the data acquisition, the tail was positioned in a tighter region of the sponge.

## 5 Monitoring System

**FIGURE 4:**
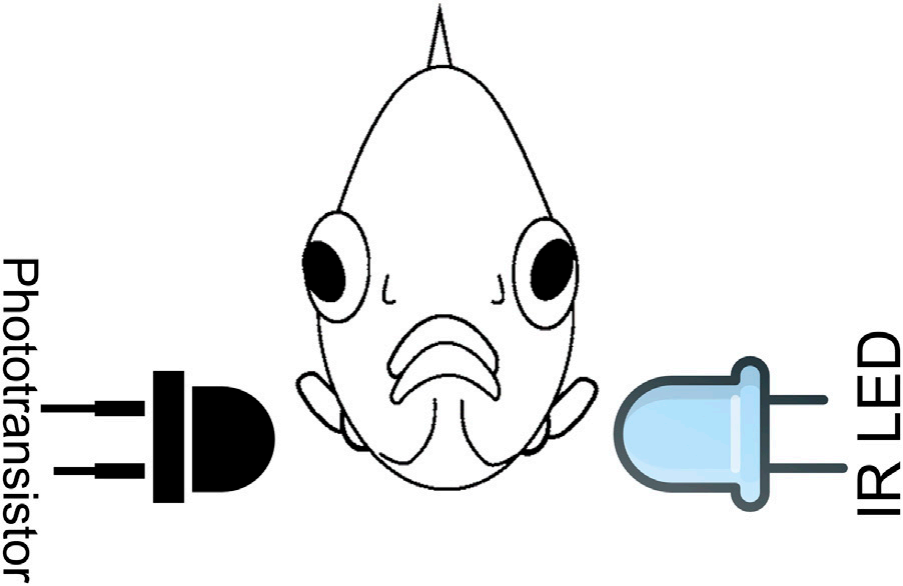
Sensor arrangement relative to the zebrafish (front view). Adapted from (Yoshida, Hirano and Shima, 2009).

The heartbeat monitoring system consists of a transmitter, an infrared light-emitting diode (IR LED), and a receiver based on a phototransistor (optical sensor). These components were placed on opposite sides of the zebrafish body, as illustrated in Figure 4 (Yoshida, Hirano and Shima, 2009) ***.*** The IR LED emits infrared photons being partially absorbed by the fish’s body. Since the number of photons absorbed varies with the blood volume, there is a correlation between the electrical signal produced and fish heartbeat.

Besides the unit responsible for the heartbeat monitoring, it was also necessary to assemble a unit for the water temperature monitoring. The temperature sensor is based on a Negative Temperature Coefficient (NTC) thermistor from Betatherm^®^ ^1^.

**FIGURE 5:**
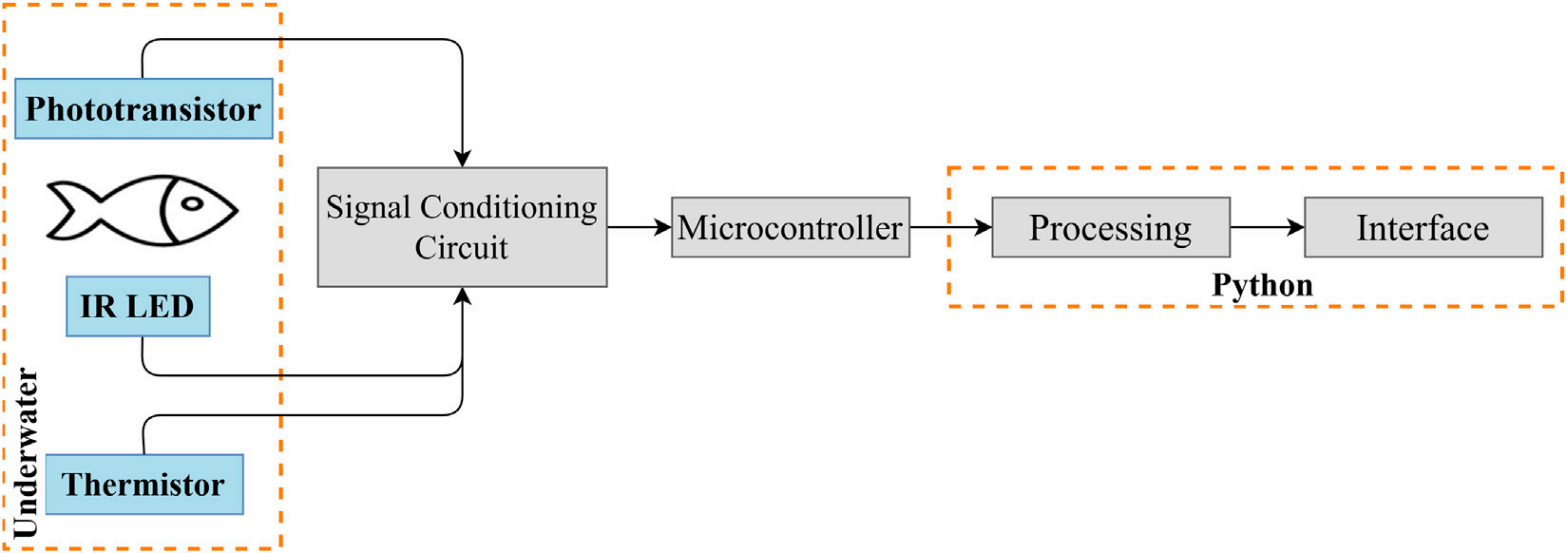
Block diagram of the heartbeat and water temperature measurement and display.

The block diagram from Figure 5 provides a functional view of the system developed, from the data acquisition stage (sensors and microcontroller) to the data processing and system control, which is executed by dedicated software. As shown in Figure 5, the sensors are underwater in the same environment as the zebrafish. First, the signal is filtered and amplified in the conditioning circuit. Then, the microcontroller (Arduino Uno R3) reads and converts the analog signal to digital and sends the data to a Python (v. 3.8) program. Finally, the data is processed and displayed in a Graphical User Interface (GUI), developed using Qt Creator (v. 4.14.1) and PyQt 5.

### 5.1 Hardware

Regarding the hardware, the heartbeat signal acquired is filtered through a simple low pass filter circuit (cutoff frequency of 2.34 Hz) and has an amplification stage (with a gain of 100). Next, the amplified signal is filtered through a high pass filter with a cutoff frequency of around 1 Hz to block the DC component and is further amplified by another amplification stage, with a gain of 45. In this way, we set the signal to be within the voltage readout range of the analog converter.

Concerning the electronic circuit that was developed to monitor the water temperature, the thermistor and a resistor (10 kΩ) were positioned in a voltage divider configuration to obtain a suitable signal for the microcontroller.

### 5.2 Firmware and Software

The microcontroller reads the signals produced by the sensors and conditioning circuits for posterior processing.

The analog value given by the water temperature conditioning circuit ( $A_{therm}$ ) is converted to voltage ( Vo ) by applying (1). The resistance of the thermistor ( $R_{Therm}$ ) correspondent to Vo is then obtained with the voltage divider formula (2). Finally, the Steinhart-Hart Equation (3) converts the thermistor’s resistance to a temperature reading (T) (in Kelvin), which is converted to the Celsius degree scale.

$$\mathrm{Vo}=5\times \frac{A_{\mathrm{therm}}}{1023}$$ (1)

$$R_{\mathrm{Therm}}\;=R1\;\times \;\frac{V_{\mathrm{in}}-V_{o}}{V_{o}}=10\;\mathrm{k\Omega}\;\times \;\frac{5-\mathrm{Vo}}{\mathrm{Vo}}$$ (2)

$$\frac{1}{T}=C1+C2\times \ln\left(R_{\mathrm{Therm}}\right)+C3\times {\left(\ln\left(R_{\mathrm{Therm}}\right)\right)}^{3}$$ (3)

where R1 is the resistor of the voltage divider circuit, $V_{in}$ is the voltage supply of the circuit, and C1 , C2, and C3 are the thermistor’s Steinhart-Hart coefficients.

The obtained temperature data and the analog readings from the heart rate conditioning circuit are then sent via serial communication (USB) from the microprocessor to the computer. This data is further processed in a dedicated software and displayed in a graphical interface based on multithreading and multiprocessing programming, enabling the processing and displaying of the data acquired in real-time.

**FIGURE 6:**
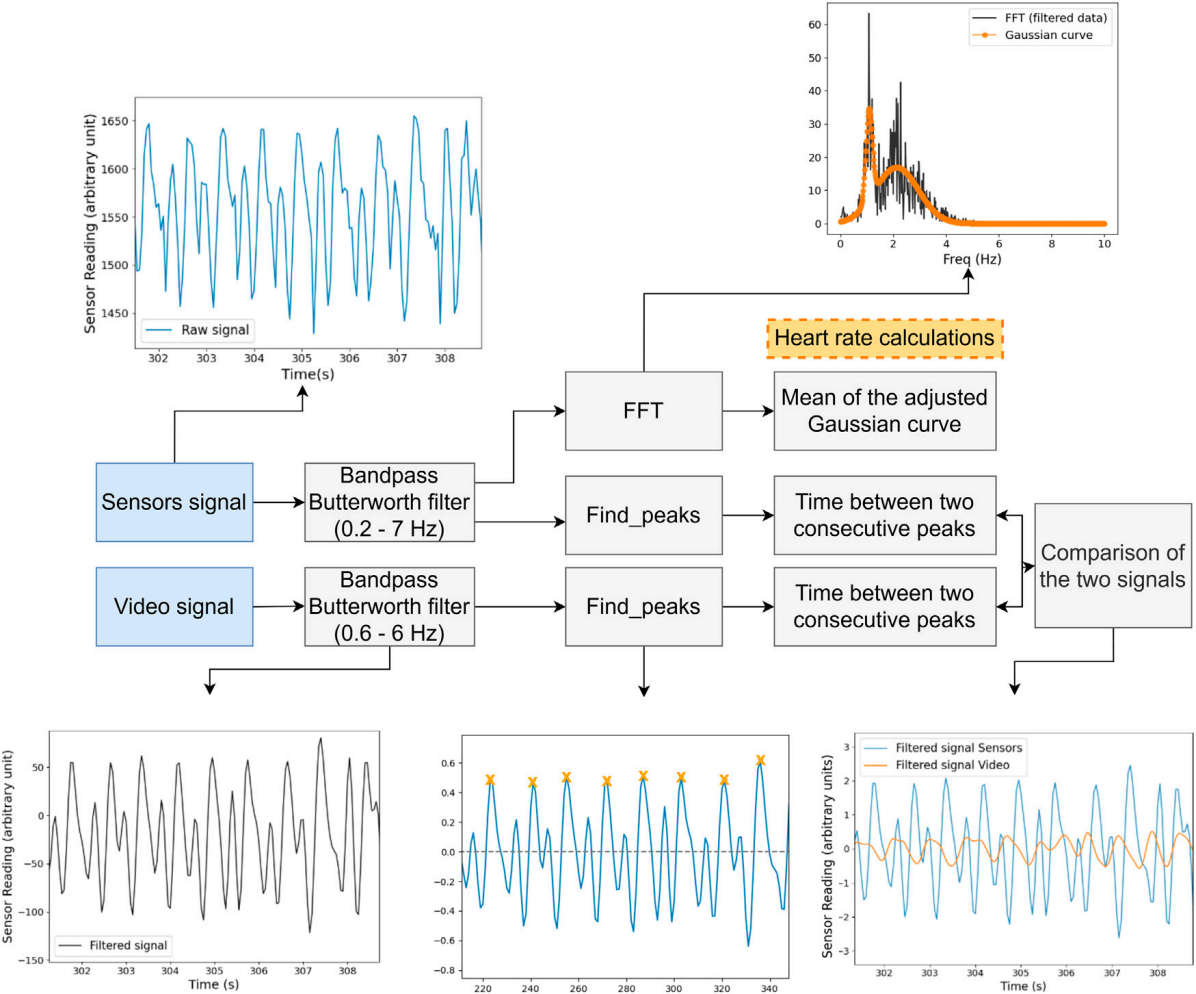
Block diagram of the analysis applied to the acquired signals, both by the sensors and the video.

The heartbeat raw data sent to the computer is filtered using a bandpass Butterworth filter, with cutoff frequencies of 0.2 and 7 Hz (Figure 6). In this way, both the signal’s DC component and higher noisy frequencies are eliminated. The heart rate calculations were based on a *find_peaks* Python function (Scipy library) (scipy.signal.find_peaks—SciPy v1.7.1 Manual, no date) that finds the local maxima inside a signal by comparing neighboring values. With the position of the peak information, it was possible to extract the time between two consecutive peaks (*delta_t*), and thus, possible to know the number of beats per minute ( bpm =60/delta_t  ).

**FIGURE 7:**
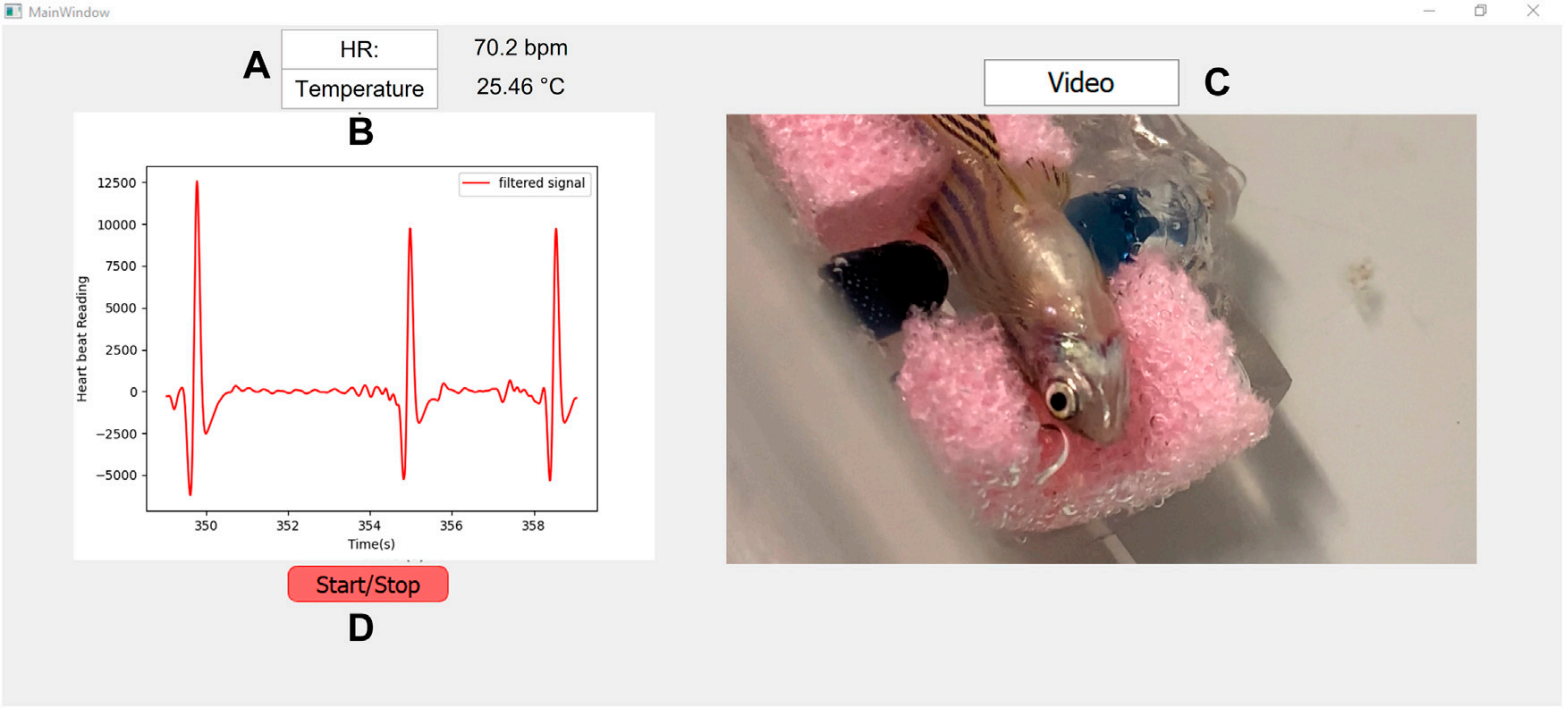
Graphical interface developed, during the signal acquisition.

Regarding the GUI design and operation, the sensor’s data are displayed in the plot area (Figure 7B). In addition, the heartbeat frequency and temperature measurements calculations are carried out parallel to the acquisition, almost in real-time, and displayed in Figure 7A.

During acquisitions, a live video of the zebrafish enclosure is also displayed for a better examination of the fish welfare (Figure 7C). The video is also recorded and synchronized with the heartbeat signal for further processing using Computer Vision algorithms.

**FIGURE 8:**
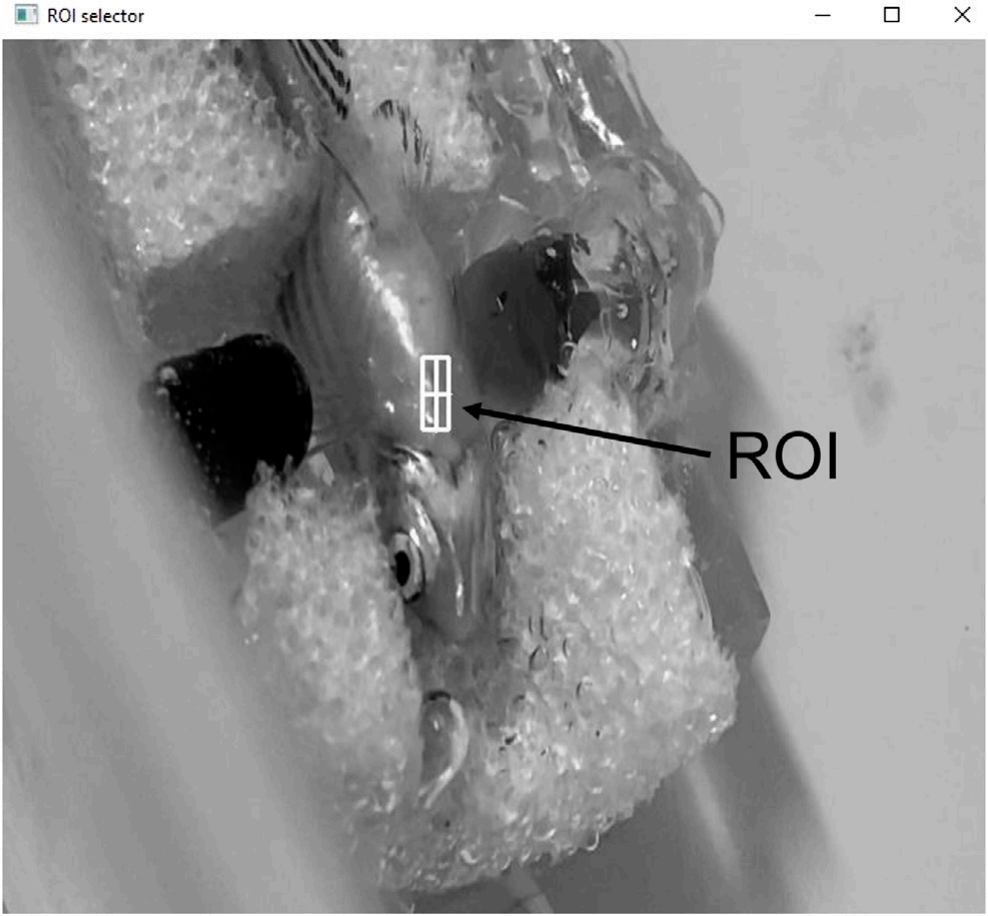
Interface of the computer vision software and ROI selection.

The video recordings were used to validate the results obtained by the sensors. The video analysis basic principle is based on the evident variation of pixels intensity value when zebrafish heartbeats. Therefore, the algorithm developed converts the frames from the video recordings to grayscale (pixel’s value varies between 0 and 255) and allows the user to choose a Region of Interest (ROI) in the image (Figure 8).

The average pixels intensity value inside the ROI is calculated for each individual video frame and represented as a time series, showing the signal corresponding to the heartbeat. After, the signal is processed and analyzed similarly to the method performed for the sensors’ signals.

## 6 Results and Discussion

### 6.1 Dissolved Oxygen and pH Measurements

The water parameters - dissolved oxygen and pH - were measured in a recipient with a similar volume to the developed enclosure. By monitoring these parameters over time, we checked that the zebrafish remains stable along with its well-being guaranteed for approximately 20 min. pH and dissolved oxygen were monitored over time since these two parameters are regularly checked in zebrafish tanks. A multi-parameter system (*Multi 3410*) was used with digital sensors, one for pH (*SenTix® 940*) and another for dissolved oxygen (*FDO® 925*).

Zebrafishes were anesthetized according to the protocol defined in Section 3 and transferred to a close recipient, free of air, with a similar volume of the zebrafish enclosure to be used. pH and dissolved oxygen concentration were registered every 30 seconds to evaluate how these values change over time.

**FIGURE 9:**
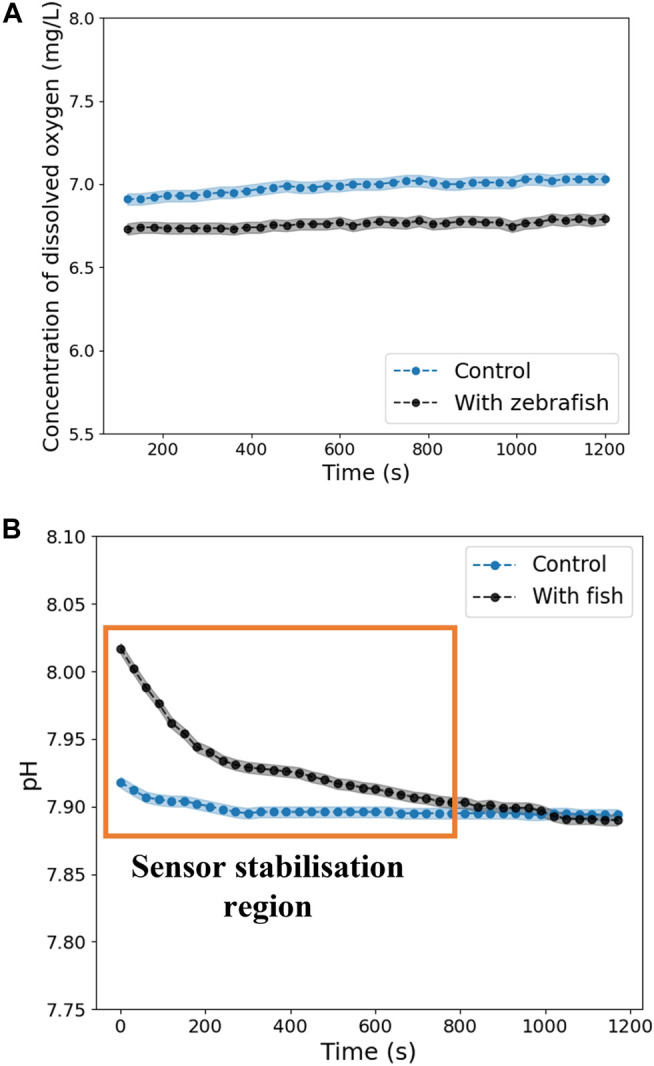
Variation of the **(A)** concentration of dissolved oxygen and **(B)** pH over time with zebrafish and without (control).

Figure 9A shows that the dissolved oxygen concentration remains practically constant even with the zebrafish presence. Relative to the pH graph (Figure 9B), it is possible to verify that the measurement takes time to stabilize. However, after that period, it is observed that the pH follows the tendency of the control study. Both graphs evidence that the zebrafish does not significantly change the environment concentration of dissolved oxygen and pH, maintaining these values within acceptable values throughout the experiments, between 6–8 mg/L (Hammer, 2019) and 6–8 (Avdesh et al., 2012), respectively.

Considering the results obtained, it was concluded that it is possible to perform PET exams on zebrafish in a closed volume without increasing the system’s complexity by including a water renewal system.

### 6.2 Water Temperature Measurements

**FIGURE 10:**
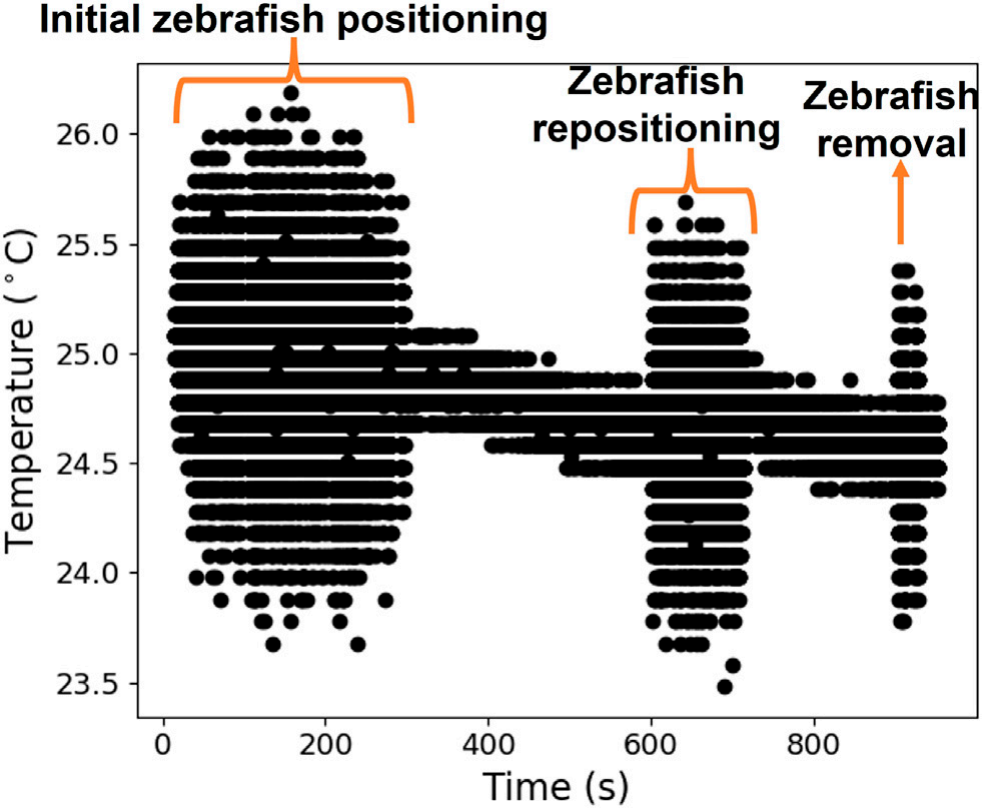
Water temperature evolution during a zebrafish trial.

The water temperature was monitored during the zebrafish’s vital signs measurements. The water temperature throughout the experiments varied between 24.5°C and 25.5°C, as shown in Figure 10. These values are within the expected and optimal range for zebrafish (24–29°C) reported in the literature (Avdesh et al., 2012; Hammer, 2019). During the vital signs measurements, it was necessary to adjust the fish positioning, which generated some movement in the water, leading to rapid changes in the thermistor response, as evidenced in Figure 10.

### 6.3 Validation of the Vital Signs Monitoring System

Before testing the vital signs monitoring system developed in zebrafish, it was decided to validate it first in human volunteers.

For this purpose, a commercial heart rate meter (Oximeter MD300C29, ChoiceMMed) was used to compare its results with the proposed system. Volunteers were asked to put one finger on the commercial heart rate meter, and another finger from the other hand between the IR LED and the phototransistor. The volunteers remained in this position for about 2 minutes, and the pulse rate was registered from both the heart rate meter and sensors every 10 seconds.

**FIGURE 11:**
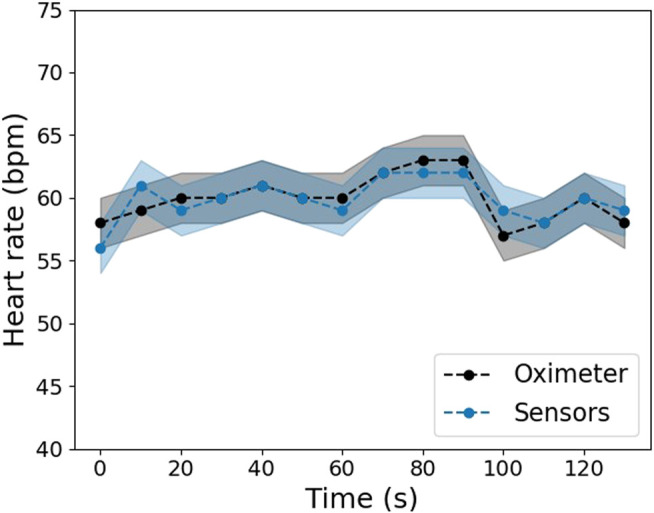
Comparison of the heart rate values obtained by the developed system's sensors and a commercial heart rate meter in one volunteer.

The monitoring system’s response is illustrated in Figure 11. The sightly changes of the heart rate values over time observed in the graph can be due to some agitation or stress of the volunteers caused by the measurement *per si*. Besides, the differences between the sensors and the commercial meter may be because it is more challenging to position the finger without moving it in the developed system. This movement can induce changes in the sensor measurements. Nevertheless, despite a 3% of the maximum difference, the heart rate values calculated by the developed system are within the error range of the commercial heart rate meter, which indicates the correct functioning of the developed system.

### 6.4 Zebrafish Vital Signs Measurements

Zebrafish vital signs measurements were performed in the Biology Department bioterium of the University of Aveiro, using the developed enclosure. Four data samples are presented in Table 1 for demonstration purposes once the remaining adult zebrafish samples contain similar information.

**TABLE 1:** Identification of the zebrafish.

	Zebrafish			
Parameters	1	2	3	4
Weight (mg)	774	800	740	380
Gender	Female	Female	Male	-
Recovered anesthesia	Yes	Yes	Yes	No

The raw data acquired for each zebrafish was done during 30 min and were filtered and processed according to the procedure described in Section 5 (Figure 6).

**FIGURE 12:**
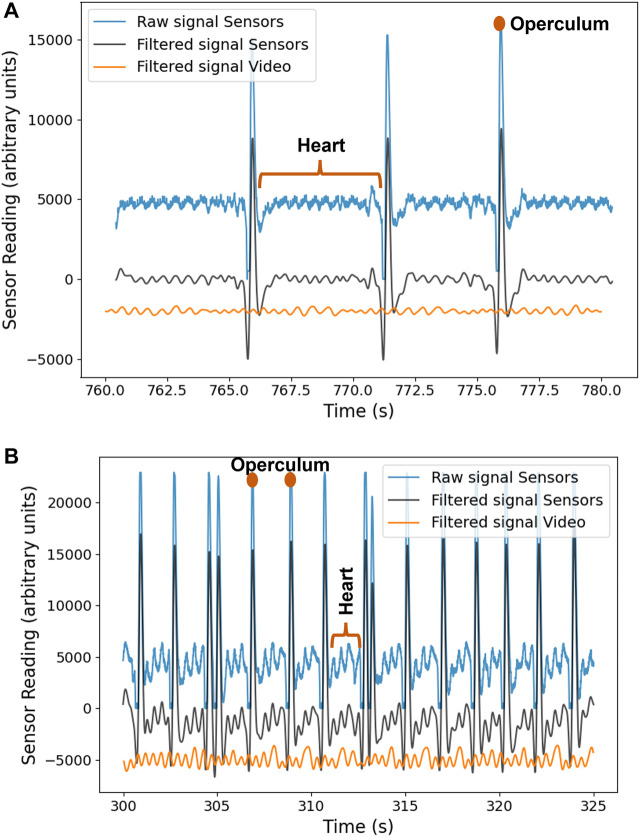
**(A)** Signal acquired from zebrafish 1 **(B)** Signal acquired from zebrafish 2.

For each fish, the heart rate obtained by the sensors was compared with the value calculated using the developed computer vision algorithm for zebrafish heart rate detection. The raw and filtered signals obtained from two zebrafish are shown in Figure 12. Both graphs make it possible to distinguish between the peaks that correspond to the heartbeat (peaks with lower amplitude) and the opercular aperture (peaks with higher amplitude). The only difference between the two signals is that the opercular aperture of the zebrafish 2 (Figure 12B) has a bigger frequency, as verified in Table 2.

**TABLE 2:** Zebrafish heart and operculum rates obtained with the sensors and video analysis method.

Zebrafish	Fig	Signal	Sensors (bpm)	Video (bpm)
1	Figure 12A	Heart	120 ± 10	127 ± 10
		Operculum	12 ± 3	12 ± 2
2	Figure 12B	Heart	88 ± 5	93 ± 2
		Operculum	32 ± 4	33 ± 5
3	—	Heart	118 ± 4	117 ± 2
		Operculum	41 ± 11	49 ± 15
4	—	Heart	115 ± 15	—
		Operculum	Nonexistent	Nonexistent

Comparing the signals obtained by the sensors and the video (Figure 12), it can be observed that some peaks are not perfectly synchronized, which is likely due to limitations of the video method (incorrect ROI selection, fish movements, or light reflections), as the SNR is lower for that method. Besides, the synchronization method is manually tuned and not done automatically by the software, contributing to a not perfect synchronization. However, despite that, the results between the two methods are in good agreement, as summarized in Table 2.

Analyzing Table 2, it is observed that the zebrafish heart rate varies between 88–127 bpm, and the operculum rate varies between 12–49 bpm. Besides, it is noticeable that both operculum and heart rate values are similar within errors when measured with the sensors’ signal or with the video; the more significant difference between the two methods found is only 8 bpm.

Regarding zebrafish 4, it was impossible to perform a video analysis due to undesired camera movements. Zebrafish 4 revealed a particular behavior since the opercular aperture was nonexistent at the end of the acquisition, which implies that the signal only contained the heartbeat pulse. Therefore, despite the zebrafish’s visible heartbeat on the acquired signal, the nonexistence of the opercular aperture indicated that the zebrafish entered into the overdose anesthesia level, leading to a non-recovery from the anesthesia even after being placed in freshwater.

Summarily, by the analysis of the graphs and Table 2, it is observed that the two methods of heart rate determination are consistent. In addition, it is possible to detect the heart rate and the presence of opercular movement, both indicators of the zebrafish’s well-being.

The obtained heart rate values compare favorably to the values found in the literature (Pereira et al., 2015; Wang et al., 2017; Santoso et al., 2020), validating our results. Nevertheless, some other discrepancies in the literature can be pointed out (Pereira et al., 2015; Wang et al., 2017; Santoso et al., 2020). For example, the different protocols and environmental conditions could justify minor discrepancies between the literature studies and the present work.

## 7 Conclusion

A suitable enclosure for *in vivo* imaging of adult zebrafish, incorporating a heart rate, opercular movement, and water temperature monitoring system, was successfully developed and implemented. As a result, the zebrafish were successfully maintained and recovered, without renewing water, for up to 30 min, the typical time for *in vivo* medical image acquisition.

The heartbeat signals obtained in zebrafish trials were in accordance with results from the video analysis, using computer vision methods. The maximum difference noticed between both methods was within the errors of the developed system.

The heart rate values obtained were between 88 and 127 bpm, which agrees with the values reported in the literature (63–125 bpm) (Wang et al., 2017).

Monitoring the opercula aperture could give information about the zebrafish’s well-being since the nonexistent opercular movement means that the zebrafish is entering the overdose anesthesia level. The developed system was able to detect the opercula aperture failure and correlate this effect with a posterior non-recovery from anesthesia, demonstrating the capability to predict critical injuries of the animal and prevent them in time.

The developed system has shown promising results presenting a challenging but interesting solution to be applied in different preclinical imaging modalities and to perform different studies combining zebrafish models and *in vivo* medical imaging.

## Data Availability

The original contributions presented in the study are included in the article/Supplementary Material, further inquiries can be directed to the corresponding author.

## Footnotes

1 BetaCurve Interchangeable Thermistor 10 kΩ with an ${\text{α}}_{25}$ of -4.39%/°C.
